# A standardized protocol for modified platelet-rich plasma collection for the treatment of interstitial cystitis/bladder pain syndrome

**DOI:** 10.14440/bladder.2024.0052

**Published:** 2025-03-14

**Authors:** Qiangping Zheng, Peng Zhang, Wei Guo, Jianzhong Zhang, Fan Zhang, Changran Ma, Yuanru Yang, Liyan Cui, Yuling Wu, Lei Zhang

**Affiliations:** 1 Department of Urology, Beijing Chaoyang Hospital, Capital Medical University, Beijing 100020, China; 2 Department of Transfusion, Beijing Chaoyang Hospital, Capital Medical University, Beijing 100020, China

**Keywords:** Intravesical injection, Blood cell separator, Platelet-rich plasma, Interstitial cystitis, Bladder pain syndrome, Treatment

## Abstract

**Background:**

Interstitial cystitis/bladder pain syndrome (IC/BPS) is a chronic and diagnostically challenging condition with limited treatment options and poor prognosis. Platelet-rich plasma (PRP) is a potential treatment for IC/BPS. Nonetheless, conventional preparation methods are inefficient, necessitating an improved approach.

**Objective:**

To design an effective and safe protocol for collecting PRP for intravesical injections for the treatment of IC/BPS.

**Methods:**

We evaluated the feasibility of the PRP collection protocol from 17 patients. After venipuncture, blood was processed using a blood cell separator containing saline and anticoagulant. One milliliter of PRP was retained for platelet concentration analysis. Each patient received six PRP injections, and their clinical data were taken before and after the procedure to assess efficacy.

**Results:**

The mean platelet enrichment coefficient was 5.11 ± 1.27, the mean flow rate was 33.19 ± 6.77 mL/min, the mean collection volume was 125.13 ± 17.49 mL, and the mean collection time was 73.69 ± 10.17 min. Eleven patients required deep venous catheterization because superficial venipuncture failed. No adverse reactions were observed during collection, and all other blood components were retained. Three patients completed six injections, four received five injections, three received four injections, one received three injections, two received two injections, and four received one injection. After treatment, 12 of 17 patients showed symptom improvement on the Global Response Assessment scale (≥5), and all demonstrated statistically significant improvements in terms of O’Leary-Sant and Visual Analog Scale scores against baseline. One patient, however, developed gross hematuria after the fourth injection.

**Conclusions:**

The proposed PRP collection protocol is relatively safe and effective. Preliminary data suggest that intravesical PRP injection is a promising treatment for IC/BPS.

## 1. Introduction

Interstitial cystitis/bladder pain syndrome (IC/BPS) represents a special type of aseptic cystitis. The symptoms include aggravated frequency, urgency, and dysuria when the bladder is full, but they improve after urination. IC/BPS differs from common bacterial cystitis in that it has a high misdiagnosis rate and poor prognosis, with general oral antibiotics proving ineffective. Conventional treatment methods for IC/BPS include oral medications, intravesical instillation of hyaluronic acid, cystoscopic hydrodistention or mucosal electrocautery under anesthesia, intravesical botulinum toxin A injection, and sacral neuromodulation. However, none of these therapies yields durable efficacy.^1^ In addition, a minority of patients may develop bladder fibrosis due to protracted chronic inflammation of the bladder tissue. These drawbacks can lead to a significant decline in compliance, exacerbating frequency and pain. In some cases, patients may require radical cystectomy and urinary reconstruction to alleviate severe pain. While this operation can improve pain relief, it affects post-operative life quality and may lead to further complications. This type of surgery is not routinely performed in urological practice, and the uncertainty of post-operative efficacy significantly increases medical risks. Managing post-operative residual pain and quality of life (QoL) remains a substantial challenge. Therefore, new treatment options are needed to manage the lower urinary tract symptoms associated with IC/BPS.

Platelet-rich plasma (PRP) is an autologous platelet concentrate that contains a concentration of platelets higher than baseline levels, along with a variety of growth factors that promote cell proliferation and differentiation, thus promoting tissue repair. Research in several countries has demonstrated that PRP was therapeutically effective in treating bone and muscle injuries,^2^ neuropathic pain,^3^ IC/BPS,^4^ and recurrent urinary tract infections.^5^ However, most studies used manual preparation methods for PRP,^1^,^5^-^7^ which require double centrifugations. This protocol necessitates the collection of PRP for each injection, and other blood components cannot be returned to the patient, resulting in significant waste. Moreover, it carries a risk of contamination,^8^ and the treatment process can be complicated. To address the limitations of this conventional method, our center has adopted a new and improved PRP collection technique and standardized its preparation protocol. From January 2023 to July 2023, we completed PRP collection and intravesical injection in 17 patients. The protocol is detailed as follows.

## 2. Materials and methods

### 2.1. Patient recruitment

A total of 17 patients clinically diagnosed with IC/BPS were involved in the study (ClinicalTrial.gov: ChiCTR2400084068). These patients included two male patients and 15 female patients, aged 19 – 67 years, with a mean age of 37 years. One patient had diabetes, whereas the others had no underlying diseases. The disease duration in the 17 patients spanned from 1 to 13 years, with a mean of 4.35 ± 2.43 years. The diagnosis of IC/BPS was confirmed based on characteristic symptoms and cystoscopic findings, such as glomerulations, petechiae, or mucosal fissures observed after hydrodistention. The inclusion criteria were as follows: (1) Patients aged over 18 years, (2) diagnosed with non-Hunner’s^6^ IC/BPS, (3) underwent cystoscopic hydrodistention under anesthesia, (4) willing to receive a total of six intravesical injections throughout the treatment process, (5) able to sign the relevant, informed consent form, and (6) those whose symptoms had not improved after undergoing at least three different treatments, including but not limited to lifestyle changes, intravesical instillation of hyaluronic acid, botulinum toxin A injection, and cystoscopic hydrodistention under anesthesia.^6^ Exclusion criteria included: (1) Platelet counts below 100 × 10^9^/L, (2) hemoglobin levels below 10 g/dL,^9^ (3) severe primary diseases (such as heart, cerebrovascular, digestive, and endocrine system disorders), (4) mental disorders, and (5) the use of non-steroidal analgesics within 2 weeks before the study. This study was approved by the Ethics Committee of Beijing Chaoyang Hospital, affiliated with Capital Medical University (Institutional Review Board [IRB] number: 2023-Science-445).

### 2.2. Related equipment and auxiliary materials

**Figure 1:**
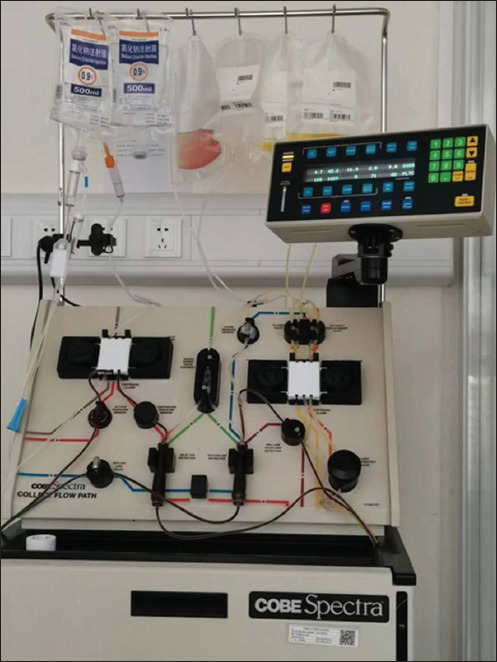
COBE Spectra blood cell separator

The COBE Spectra blood cell separator (Terumo BCT, Inc., USA; Figure 1) is functionally capable of platelet collection, removal, and plasma exchange, among others. During the procedure, we input the subject’s gender, height, weight, blood cell volume, and other information, allowing the system to automatically calculate the total blood volume, total transport volume, and the amount collected. The sodium citrate-glucose solution, composed of sodium citrate, citrate acid, and glucose, was used for PRP preservation after collection. A 500 mL saline solution was employed to fill and empty the air in the pipeline, thereby preventing air embolism.

### 2.3. The modified and standardized platelet-rich plasma collection protocol

#### 2.3.1. Preparation before platelet-rich plasma collection

All patients were required to undergo a routine examination on the day of admission, with particular attention paid to platelet and hemoglobin levels. Once the blood test results were available, the platelet concentration, height, and weight of each patient were recorded. The Blood Transfusion Department was then consulted to assess the feasibility of blood collection. The necessary materials, including two 500 mL bags of saline, anticoagulant, disinfection consumables, sterile accessories, infusion connectors, and a 16 – 18G indwelling steel needle, were in place. To prevent hypocalcemia from citrate-induced acidosis during blood collection, calcium gluconate was also available for infusion, if necessary.^10^

#### 2.3.2. Platelet-rich plasma collection

**Figure 2:**
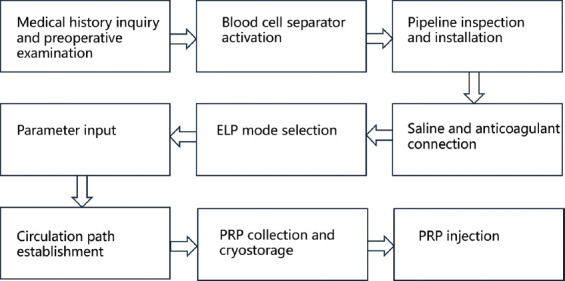
PRP collection and therapy protocol Abbreviations: ELP: Erythrocyte, leukocyte, and platelet; PRP: Platelet-rich plasma.

A designated clinician and nurse assisted with the patient’s venipuncture. If superficial veins in the upper limb were difficult to puncture with a 16G needle, or if the required blood collection rate could not be achieved, the procedure would be switched to a deep jugular vein puncture for PRP collection. The physician responsible for the blood collection equipment then started the blood cell separator, verified and installed the disposable tubes, and attached 500 ml of normal saline and sodium citrate-glucose solution as an anticoagulant. The erythrocyte, leukocyte, and platelet (ELP) mode was selected, and patient parameters – including gender, height, weight, hematocrit, and platelet count – were entered. After successfully establishing the patient’s circulation circuit, the instrument began the PRP collection protocol. During collection, the clinician closely monitored the patient’s condition to address any adverse events promptly (Figure 2). After collection, the patient was returned to the ward and given an infusion. The total PRP obtained was divided into six bags: one was used for intravesical injection on the same day, and the remaining five bags were stored at −80°C for subsequent use.

#### 2.3.3. Platelet-rich plasma injections

The patient was placed under general anesthesia and positioned in the lithotomy position. After routine iodophor perineal disinfection and draping, an F21 cystoscope was inserted. The bladder was infused with saline at a pressure of 80 cmH_2_O to achieve adequate hydrodistension. Subsequently, a bladder injection needle was introduced, and approximately 30 evenly distributed sites were selected. About 0.5 mL of PRP was injected at each site, with an injection depth of approximately 1 mm. The injections were systematically performed from left to right and top to bottom to avoid repeating injections at the same site. After the procedure, a urinary catheter was left in place for one day, and a single dose of intravenous antibiotics was administered to prevent urinary tract infections.

### 2.4. Data collection

The circulating blood volume, total anticoagulant amount, blood collection flow rate, PRP collection volume, collection time, adverse reactions, and corresponding treatments were recorded. Approximately 1 mL of the harvested PRP was retained and sent to the laboratory for platelet concentration determination on a blood cell analyzer. Post-operative efficacy was assessed 1 month after each injection on the Global Response Assessment (GRA) scale. A GRA score of ≥5 was considered an improvement, while a score of ≥6 was indicative of a good outcome. A GRA score of ≤4 indicated ineffectiveness. The following parameters were recorded both preoperatively and postoperatively: daily urination frequency, nocturia frequency, functional bladder capacity, O’Leary-Sant scores, Visual Analog Scale (VAS) scores for pain, QOL scores, as well as pelvic pain, urgency, and frequency scores.

### 2.5. Statistical analysis

Data analysis was performed using SPSS 20.0 software (International Business Machines Corporation, USA). Data were presented as mean ± standard deviation (SD). Paired *t*-tests were used to compare pre- and post-treatment data, with a significance level set at α = 0.05. A *p* < 0.05 was considered statistically significant; otherwise, it was considered not significant.

## 3. Results

A total of 17 patients clinically diagnosed with IC/BPS, requiring intravesical PRP, were included in this study. All patients successfully underwent PRP collection without any adverse events during the process. The PRP was divided into six bags, with each bag containing approximately 20 mL. One bag of PRP was used for intravesical injection on the same day, and the remaining five bags of PRP were stored at −80°C for subsequent injections. The mean platelet enrichment coefficient in PRP was 5.11 ± 1.27. The mean blood collection flow rate was 33.19 ± 6.77 mL/min, and the mean PRP collection volume was 125.13 ± 17.49 mL. The mean collection time was 73.69 ± 10.17 min (Table 1). Eleven patients required deep jugular vein puncture due to failed superficial vessel puncture. No additional blood component loss occurred during collection and PRP was safely transfused back to all patients. No adverse reactions were reported during the blood collection procedure. Three patients completed six PRP bladder wall injections, four patients received five injections, three received four injections, one received three injections, two received two injections, and four received one injection.

**Table 1:** General data before and after platelet-rich plasma collection

PI	Gender	Age	BMI	PC	DD	EC	BCR	PCA	CT	AE	RM
1	Male	23	20.48	252	2	3.15	-	-	-	No	No
2	Female	32	21.05	242	2	4.08	26	117	70	No	No
3	Female	67	23.71	252	5	2.37	42.5	145	63	No	No
4	Female	44	21.78	256	2	5.23	26	136	73	No	No
5	Female	19	25.34	239	6	5.34	40	138	70	No	No
6	Male	40	30.78	242	10	7.03	42.5	130	65	No	No
7	Female	57	25.91	210	6	3.97	37.5	157	67	No	No
8	Female	27	21.50	304	1	4.33	21	108	90	No	No
9	Female	39	22.05	210	1	4.76	30	141	85	No	No
10	Female	60	28.40	185	4	4.95	40.5	141	65	No	No
11	Female	23	22.04	150	1	4.91	28.5	116	100	No	No
12	Female	24	20.90	234	2	4.85	32	109	77	No	No
13	Female	63	23.88	151	5	5.72	41.5	101	77	No	No
14	Female	27	20.06	216	8	6.07	32.5	114	70	No	No
15	Female	22	17.38	299	2	4.17	33	115	65	No	No
16	Female	33	19.84	232	13	7.38	29.5	136	70	No	No
17	Female	26	18.37	221	4	6.62	28	98	72	No	No

Notes: Age and DD are in years; BCR is in mL/min; CT is in minutes; PC is in *10^9^/L; PCA is in mL. Abbreviations: AE: Adverse events; BCR: Blood collection rate; BMI: Body mass index; CT: Collection time; DD: Disease duration; EC: Enrichment coefficient; PC: Platelet concentration; PCA: Platelet-rich plasma collection volume; PI: Patient identification; RM: Responding measures.

Since this study was a preliminary exploratory investigation, some patients have not yet completed the full course of treatment. To ensure comparability and consistency of the results, we defined 1 month after each patient’s last PRP injection as the study endpoint. Clinical indicators before treatment and at the endpoint were compared to evaluate the preliminary efficacy of PRP therapy. After their final injection, 12 out of 17 patients showed symptom improvement (GRA ≥5), resulting in an overall response rate of approximately 70.59%. Statistically significant improvements in O’Leary-Sant and VAS scores were observed in all 17 patients after treatment compared to baseline (*p* < 0.05), while no significant changes were found in other indicators (Table 2 for detailed results).

**Table 2:** Comparison of clinical data before surgery and 1 month after the last injection

Clinical indicator	Baseline	One month after the last injection	*p*-value
Daily frequency	13.59±7.95	13.06±7.22	0.644
Nocturia frequency	3.41±3.34	2.71±2.66	0.097
Functional bladder capacity	200.88±78.49	211.47±91.24	0.683
O’Leary-Sant score	22.12±8.03	16.00±7.77	0.001
PUF score	15.94±6.08	14.65±6.61	0.407
VAS score	4.09±3.03	2.35±2.42	0.004
QOL score	4.47±0.94	3.59±2.29	0.156

Abbreviations: PUF: Pelvic Pain, Urgency, and Frequency; QOL: Quality of life; VAS: Visual Analogue Scale.

One patient developed gross hematuria after the fourth injection, but this resolved after four days of bladder irrigation, and she was discharged without further complications. The blood collection and injection process for the remaining patients were completed successfully.

## 4. Discussion

Platelet-rich plasma therapy has been utilized in various medical fields for over 30 years,^11^ including orthopedics, dermatology, and ophthalmology. However, the full biological composition of PRP remains unclear. The fundamental rationale behind its use is to provide an array of growth factors and cytokines that promote tissue healing, thus creating an optimal molecular microenvironment in which regenerative mechanisms can work synergistically.^12^ PRP is particularly rich in α-granules of platelets, which contain a multitude of growth factors, including platelet-derived growth factor, transforming growth factor β, and insulin-like growth factor.^13^ These bioactive molecules can stimulate the growth and differentiation of cells in the targeted tissues. Among the numerous pathophysiological theories of IC/BPS proposed, a key factor is the dysfunction in the bladder mucosa. Specifically, an inability to effectively repair injury to the bladder lining leads to increased mucosal permeability, triggering a cascade of physiological changes and clinically causing symptoms. This theory is supported by multiple studies. The central idea behind PRP therapy is to promote the repair and regeneration of damaged tissues. For IC/BPS patients, PRP injection therapy differs from traditional treatment methods in that it is potentially a more radical and curative approach. The pathogenesis of IC/BPS is intimately related to bladder wall inflammation and defects in the urothelial barrier. The cytokines present in PRP have been shown to promote mucosal repair and suppress inflammation. In contrast, inflammation in Hunner’s IC/BPS, which involves Epstein–Barr virus^14^ infection in ulcerative lesions, is currently treated mainly with bladder mucosal cauterization. Therefore, this study focused exclusively on non-Hunner’s IC/BPS patients.

The modified PRP collection technique used in our center involves the preparation of PRP with a blood cell separator,^8^ and is therefore different from the conventional PRP collection methods reported in the literature. Based on the current exploratory study and previous research^8^,^15^ we have preliminarily identified several advantages of this blood collection strategy: (i) The risk of PRP contamination is reduced, (ii) the platelet concentration is higher, (iii) there is less loss of the patient’s own blood, and (iv) unwanted blood components can be transfused back into the patient. In this study, the COBE Spectra blood cell separator was used to segregate blood components in a sterile, fully closed system, with the remaining blood components being recycled back into the patient’s body. In contrast, the conventional PRP preparation method involves external centrifugation of whole blood to isolate useful components, discarding the remaining blood components. Our new PRP preparation method not only significantly lowers the risk of infection but also sets the blood collection machine parameters based on the patient’s physical condition, thus minimizing the loss of other blood components and reducing the likelihood of adverse events. Zhang *et al*. compared the blood cell separator method to its conventional manual preparation counterpart^15^ and concluded that the former outperforms the latter in terms of preparation time, platelet enrichment coefficient, and residual rates of red and white blood cells.

Several factors, including patient age, centrifugal force, rotational speed, and hematocrit, affect the platelet concentration in the prepared PRP.^16^ Using the blood cell separator allows for tailoring selection parameters to individual patient conditions, yielding the desired platelet concentration. The enrichment coefficient, defined as the ratio of platelet concentration in PRP to that in whole blood,^8^ is typically recommended to be 4 – 6 times higher than that of whole blood.^17^ In this study, the average platelet enrichment coefficient was 5.11 ± 1.27, which aligns well with clinical therapy requirements. Therefore, the platelet concentration of our PRP clinically meets the therapeutic standards, providing a solid foundation for subsequent therapeutic efficacy in IC/BPS.

The modified PRP collection protocol not only reduces patient trauma but also simplifies the diagnostic and therapeutic protocols, thereby reducing the financial burden involved. In a study on large-scale PRP production, each PRP collection was cryopreserved at −40°C within 24 h after collection for up to 2 years. After testing, 14 out of 553 samples were discarded, indicating that frozen PRP retains its quality and features over time.^18^ By utilizing the blood cell separator to control the PRP collection protocol, we were able to complete the entire PRP collection for the treatment, dividing the PRP evenly into six small bags. Except for one bag used in the intravesical procedure on the same day, the remaining five bags were stored in a −80°C refrigerator for subsequent intravesical injections within 5 months. This approach minimized additional injury to patients during subsequent PRP injections and significantly improved the PRP protocol by reducing trauma, infection, and waiting time.

This study is still underway and, therefore, most patients have not yet completed the planned PRP injection treatment. However, preliminary data analysis showed that approximately 70.59% of IC/BPS patients accomplished symptom relief following intravesical PRP injections, as indicated by improved O’Leary-Sant and VAS scores. Based on the current results, PRP injection therapy appears to effectively alleviate symptoms in general and pain in particular. Studies have shown that platelets possess analgesic effects.^19^ The specific mechanism might be related to the cytokines and growth factors contained in PRP, and we have planned to further look into this in future studies. In recent years, researchers have also adopted PRP instillation as a treatment option. This approach offers advantages such as non-invasiveness and freedom from anesthesia. However, it necessitates specific conditions for the platelet concentrate in PRP to penetrate the submucosa. PRP instillation relies on the higher permeability of Hunner lesions to better reach the diseased site and utilizes osmotic pressure differences^20^ to allow particles larger than 10 nm to penetrate the bladder barrier.^21^ Therefore, PRP injection therapy can more easily achieve the therapeutic goal.

In the preliminary exploration of the application of this modified blood PRP therapy protocol, we also focused closely on its safety aspects. Previous studies have shown that during platelet collection using the blood cell separator, various adverse reactions can occur, including hematoma, thrombophlebitis, paleness, dizziness, and citrate poisoning.^22^ Throughout the entire design of the collection protocol, we prioritized patient safety, from the initial assessment to the PRP collection and till the intravesical injection. The results of this study demonstrated that, after the standardized PRP collection protocol, no adverse events or complications took place in any of the patients, except one patient who experienced gross hematuria after surgery. It is important to emphasize that, among the first eight female patients, five required deep vein puncture for blood collection due to the difficulty in performing superficial venipuncture. The mean age of these patients was 46 years, with the body mass index (BMI) being 23.71, 25.34, 25.91, 21.50, and 28.40, respectively. This observation suggests that superficial venipuncture can be particularly challenging for female patients with a lower BMI or in advanced age. Consequently, in the subsequent blood collection protocol, we evaluated patients’ conditions in advance and opted for deep vein catheterization, whenever necessary, to minimize blood collection-related trauma. Among the 17 patients who underwent more than 60 bladder injection procedures, only one developed severe intermittent gross hematuria postoperatively. After receiving solifenacin (for easing bladder spasms) and intravesical irrigation, the patient was discharged without further complications. We believe this complication resulted from the abundant blood vessels in the bladder and severe bladder spasms, as well as the potential damage to small blood vessels during the injection procedure. The development of this complication highlights the need for careful examination of the bladder wall during the procedure. If necessary, electrocoagulation should be applied to any obvious bleeding points in the bladder during surgery. Postoperatively, urine color and hemoglobin levels should be monitored, and the hospitalization period should be extended as needed based on the patient’s condition.

This study is subject to several limitations. Our new technique has not been put into practice for long, so relevant patient data are still limited, and the advantages of this new method have yet to be fully confirmed. Moreover, the efficacy of PRP largely depends on its preparation method.^23^ This study was a single-arm prospective trial, without being compared to the conventional manual preparation method of PRP. Studies have shown that PRP works best when the concentration is 5 – 7.5 times that of whole blood.^7^ Although the platelet enrichment coefficient of our PRP met the expected requirements, most samples did not reach the optimal concentration for maximal effectiveness. To address this limitation, we will continue to refine the technique to achieve the best PRP concentration. The lack of a placebo control is another limitation of this study. Conducting a placebo-controlled trial would face significant challenges in obtaining ethical approval. The most likely source of placebo effects in this treatment is the bladder hydrodistension procedure, as hydrodistension *per se* is a well-established therapy for IC/BPS. However, the patients included in this study had all previously failed to respond to hydrodistension treatment, which minimized its potential impact on our findings. In future studies, we will include more patients and provide a more comprehensive demonstration of the safety and effectiveness of our new technique through additional data.

## 5. Conclusion

The new modified PRP collection technique, developed by our team in partnership with the Department of Blood Transfusions, has led to the establishment of a relatively mature and standardized PRP collection and treatment protocol that is typical of our practice. The PRP that satisfies the standard concentration required for clinical treatment can now be collected efficiently. Compared with conventional blood collection methods, our protocol reduces patient trauma to a greater extent, is more convenient with each PRP injection, and ensures higher safety during both blood collection and intravesical injection. The incidence and severity of post-operative complications were low. Our preliminary analysis suggests that intravesical PRP injection is both effective and safe for the treatment of IC/BPS. The implementation of these PRP collection and treatment protocols is likely to benefit more patients with IC/BPS.

## Data Availability

The datasets generated and analyzed during the current study are available from the corresponding author upon reasonable request.
